# Using Co-Occurrence to Evaluate Belief Coherence in a Large Non Clinical Sample

**DOI:** 10.1371/journal.pone.0048446

**Published:** 2012-11-14

**Authors:** Rachel Pechey, Peter Halligan

**Affiliations:** School of Psychology, Cardiff University, Cardiff, United Kingdom; Maastricht University Medical Centre, The Netherlands

## Abstract

Much of the recent neuropsychological literature on false beliefs (delusions) has tended to focus on individual or single beliefs, with few studies actually investigating the relationship or co-occurrence between different types of co-existing beliefs. Quine and Ullian proposed the hypothesis that our beliefs form an interconnected web in which the beliefs that make up that system must somehow “cohere” with one another and avoid cognitive dissonance. As such beliefs are unlikely to be encapsulated (i.e., exist in isolation from other beliefs). The aim of this preliminary study was to empirically evaluate the probability of belief co-occurrence as one indicator of coherence in a large sample of subjects involving three different thematic sets of beliefs (delusion-like, paranormal & religious, and societal/cultural). Results showed that the degree of belief co-endorsement between beliefs within thematic groupings was greater than random occurrence, lending support to Quine and Ullian’s coherentist account. Some associations, however, were relatively weak, providing for well-established examples of cognitive dissonance.

## Introduction

Although the formal study of beliefs has received comparatively little interest from the cognitive neurosciences [1], the study of false beliefs (delusions) has proved a productive field over the past decade when explaining delusions in terms of impairments to cognitive processes [2], [3]. For the most part, these studies have focused on highly specific, monothematic problematic beliefs related to misidentification and body awareness [4]. However, patients and controls claim to hold many different types of beliefs with different degrees of intensity and few studies to date have looked at the internal consistency and interrelationships between such beliefs.

This is surprising given Festinger’s influential premise outlined in 1957 [5], which proposed that as human beings we are motivated to achieve consonance (i.e. agreement) and reduce or avoid cognitive dissonance by filtering new beliefs through pre-existing schema to ensure consistency [6], [7]. Consequently “a proposition is believed (empowered to guide behaviour) when the proposition’s meaning is represented, coded or symbolized in a mental system” [8], with meaning having been defined as the coherence between beliefs and without which our actions “would be random and disconnected from our surroundings” [9].

Characterising this relationship between beliefs as an epistemological metaphor, maximizing coherence, Quine and Ullian [10] proposed the existence of a ‘web of beliefs’ as a necessary condition for beliefs to be meaningful, as well as an important process for the acceptance, rejection and integration of new beliefs. As such, the “web” metaphor provides a collective explanatory network, where changes in one part afford and impact changes elsewhere. Many contemporary philosophers favour coherence theories of knowledge [11]. Davidson [12], [13] argued that beliefs can only be understood by relating them to a background of other beliefs and desires. Fodor [14] also considered beliefs to be related to and justified by reference to other propositions. In social psychology, belief networks are considered central to many theories in the psychology of attitudes [15]. According to the coherence theory of truth, a proposition coheres with a set of propositions if it is *entailed* by other members of the set [16].

These ideas were further developed by Thagard [11], who considered a belief to be justified “not because it is indubitable or is derived from some other indubitable beliefs, but because it coheres with other beliefs that jointly support each other” (p.5). Thagard accounted for coherence in terms of constraint satisfaction, extending discussion of coherence to a much wider range of cognitions, including perception and decision-making [11]. In this manner, a mental representation such as a belief could either cohere (i.e. have a positive constraint) or not cohere (i.e. have a negative constraint) with other representations. Coherence was maximised by accepting or not accepting beliefs so as to satisfy the most constraints (both positive and negative). In this way, Thagard also left room for some incoherence between beliefs (unlike the philosophical argument of Quine and Ullian).

Problems arising from holding different beliefs can result in internal conflict or cognitive dissonance, a term coined by Festinger [5] to capture the discrepancy between active beliefs and the overriding drive for meaningful coherence. Festinger [5] proposed that people are motivated to avoid such cognitive dissonance (i.e. holding contradictory beliefs, thoughts, attitudes, etc). As such, belief coherence requires (in part) subjects having some awareness of the beliefs held and their potential for inconsistency. In the clinical literature, there are examples of somatoparaphrenia [17], [18], where probing the phenomenal condition (e.g., supernumerary phantom limb) helps reveal an uncomfortable awareness of contradictory claims. Dysfunctional belief evaluation and revision has been proposed to play a key role in the maintenance of delusions [4], and interestingly, such a deficit may be quite selective, with parts of the belief system unaffected [19]. However, whether long-term cognitive dissonance presents in the general population is unknown.

Exploration of belief coherence could provide a novel way of extending current deficit models of belief (e.g. delusions) by elucidating the effects of aberrant beliefs on the coherence (or not) between other beliefs. A cognitive neuropsychiatric approach suggests that unusual beliefs such as delusions can be best explained by understanding the normal processes by which beliefs are formed and subsequently perturbed. The exact nature of the deficit(s) that give rise to delusional beliefs is not known, nor is the framework or context in which such beliefs develop. One potential candidate factor that could be predicted to selectively compromise belief, is the coherence between belief relationships. A clinically relevant or salient delusion (e.g., Capgras) might for example “infect”, “predispose” and/or subjugate a cohort of existing beliefs, or indeed provide for new content-dependent secondary delusions (e.g., paranoia).

Despite contemporary philosophy favouring coherence theories of belief [5], [10], [11], [13] there is an absence of empirical studies. As such, the approach remains largely theoretical and it is not clear whether the ‘web’ metaphor actually provides a useful heuristic for how similar beliefs might actually cohere in reality. Indeed, “the nature of coherence is usually left vague, with no method provided for determining whether a belief should be accepted or rejected on the basis of its coherence or incoherence with other beliefs” [11: p.41].

Although the concept of coherence remains “vague”, a number of authors have begun to define the construct in probabilistic terms following the intuitive notion that coherent propositions should “hang together well” [20], and that coherence remains a matter of degree [21]. As such, one might expect that groups of beliefs that share certain characteristics may co-occur within individuals responses to belief questions due to underlying coherence between their sets of beliefs, e.g. beliefs in an afterlife may necessitate belief in a spirit separate from the body. However, co-occurrence is not the same as coherence: e.g. a belief in God may co-occur with beliefs in communication with the dead due to a common notion related to beliefs in spirits, but lack of incoherence (rather than coherence per se) might be also driving this co-occurrence. Nevertheless, the degree to which beliefs co-occur more than expected on the basis individual levels of endorsement provides one quantifiable indicator of underlying coherence between beliefs. In this paper co-occurrence between endorsed beliefs are used as a measurable form of association, the relative extent of which may indicate the relative degree of coherence. Moreover, the degree to which beliefs that would be expected to show very high levels of coherence fail to show high co-occurrence is also examined.

Preliminary support for co-occurrence between thematic groups of beliefs was reported by Pechey and Halligan [22], who showed that participants’ responses to items within a thematic category of belief (delusion-like beliefs, paranormal and religious beliefs, or societal/cultural beliefs) largely correlated highly with each other (using Cronbach’s alpha) in a large sample of non-patient subjects. Correlational analyses between categories, however, showed that while different belief types (DLB and P&RB) were significantly correlated, societal/cultural beliefs (SCB) were different and largely unrelated.

The current study builds on these findings by:

- Exploring the range of co-endorsements for distinct belief items (comprising the Cardiff Beliefs Questionnaire [CBQ]), by looking at specific belief pairs and the extent to which co-endorsements of these occur more than would be predicted given the levels of endorsement for each *(i.e. investigating whether holding a belief of a particular type increases the likelihood of holding a belief of a similar type).*
- Investigating the degree to which specific belief pairs, designed to have similar content are not both endorsed by individuals (i.e. investigating the extent to which members of the general population report dissonance between beliefs)

In particular, it was hypothesised that: (i) beliefs within thematic groups would more likely co-occur than those between groups; (ii) beliefs within the thematic groups ‘delusion-like’ and ‘paranormal and religious’ would show greater co-occurrences than those in the ‘societal/cultural’ group, given the less strong thematic associations within the latter group; (iii) that there would be greater co-occurrence between ‘delusion-like’ and ‘paranormal and religious’ beliefs than between either group and ‘societal/cultural’ beliefs, given the strong and reliable association between delusions and paranormal beliefs [23]–[26] and finally (iv) the incidence of dissonant beliefs reported by non-clinical participants would be small.

## Methods

### Ethics Statement

The study was approved by the Cardiff University School of Psychology ethics committee. All participants of the telephone interview gave verbal consent, in accordance with the protocol approved by the ethics committee.

### Participants

The responses of 1,000 adults (aged 18 years or over) were examined. A stratified random sampling technique was used to obtain a large sample from across Britain, with quotas set on age, gender and employment status. Computer-assisted telephone interviewing was carried out by an experienced market research company (MRUK), using numbers generated by random digit dialing. The number of refusals was not recorded, so an overall response rate could not be reported. Telephone interviews were chosen as being more conducive to frank responses than face-to-face interviews. Of the participants, 19.4% were aged 18–29 years, 29.2% aged 30–44 years, 24.5% aged 45–59 years and 26.9% aged 60 years or over; 52.1% were female. Socioeconomic groups (using British classifications according to occupation/prior occupation) were AB (e.g., managers, administrators and professionals; 34.6%), C1 (e.g., clerical workers, call centre agents, nursery nurses; 21.2%), C2 (technical and craft workers; 9.3%), DE (semi/unskilled manual workers; 20.3%) and not classified (14.6%).

### Cardiff Beliefs Questionnaire (CBQ)

This study reports the first detailed analysis of the probability of co-endorsement for a large number of belief items (17 delusion-like beliefs, 10 paranormal and religious beliefs, and 19 societal/cultural beliefs). All items comprised the Cardiff Beliefs Questionnaire (CBQ), which was designed to detect delusion-like beliefs in non-clinical samples. The CBQ avoids clinical vocabulary and locates questions within a broader non-clinically focused context, in order to encourage participants to endorse items in an honest and open manner. Respondents are offered 5 response options: ‘Do not believe’, ‘Don’t know’, ‘Weakly believe’, ‘Moderately believe’, ‘Strongly believe’. The CBQ has good reliability and validity [22].

Given that the choice of belief questions employed largely determines the degree of expected association (coherence) between beliefs, we describe briefly how the 3 sets of thematic questions were selected. To avoid ambiguity and conscious of time constraints, one question per sub theme was used.The set of delusion-like beliefs was designed to sample across a range of delusional themes, with one question representing each delusional theme. DLB themes were taken from DSM-IV-TR [27], existing clinical measures [28]–[31] and relevant examples from the cognitive neuropsychological research literature [32]–[34]. Paranormal questions were based on reviews of published market research polls [35]–[37] and paranormal belief measures [38]–[40]. In contrast to the DLB category, within the P&RB category, there were four belief pairings that were specifically designed to have similar content, so as to investigate belief dissonance (see Table 1 for these belief pairings). Finally the societal and cultural questions were largely based on themes from market research surveys, representing potentially controversial or topical issues, and each item was selected independently with the exception of one further pair of items designed to investigate dissonance.

**Table 1:** The percentage of selected belief pairs reported inconsistently.

Item A	Item B	Percentage of those reportingbelief in item A butnot in item B	Percentage of those reporting strong belief in item A but not in item B
Reincarnation (i.e. that when you die yoursoul is reborn in another body)	The soul or spirit survives death	41.6% (n = 159)	38.3% (n = 36)
Some people communicate with the dead	The soul or spirit survives death	22.6% (n = 91)	21.5% (n = 34)
Earth has been visited by aliens from other solar systems	Extra-terrestrial life	12.4% (n = 43)	8.3% (n = 5)
Some people are possessed by evil spirits	Demons or evil spirits	9.4% (n = 38)	4.2% (n = 4)
The theory of evolution	Humans share a commonancestor with apes	8% (n = 67)	5.6% (n = 26)
Humans share a common ancestorwith apes	The theory of evolution	7.7% (n = 64)	4.3% (n = 22)

### Analysis

The main analysis involved a group level assessment, with the aim of providing an indication of the level or extent of co-endorsement over and above those expected by chance for the total subject sample (N = 1000). The number of times each belief pair within the total set was co-endorsed was analysed using chi-square tests (with Yates’s and Bonferroni’s corrections), taking into account the levels of endorsement that each belief received separately. The phi statistic was used as a measure of degree of association. The degree to which co-occurrence was present or absent between belief pairs expected to show strong coherence, was examined. Mann-Whitney U tests were used to examine demographic differences when reporting potentially dissonant beliefs.

## Results

### 1. Coherence between Beliefs

**Figure 1:**
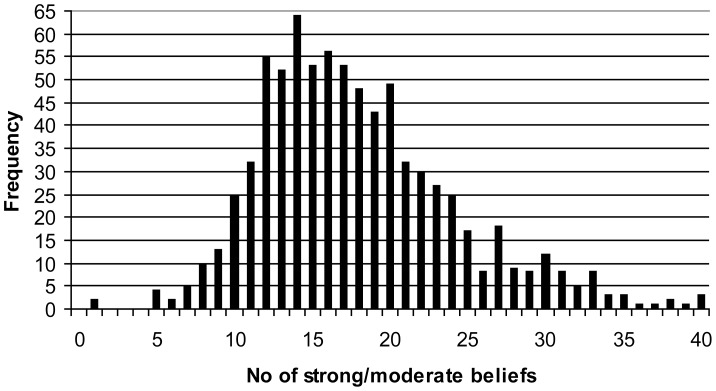
The number of strong/moderate beliefs reported (n = 1000).

To examine the degree of coherence we considered levels of co-endorsement between belief pairs within individuals using the total sample of responses to the 46 CBQ questions. Although the number of beliefs (mean = 17.6) endorsed ‘strongly believe’ or ‘moderately believe’ by subjects in the study as a whole varied (see Figure 1), with males (M = 17.2) endorsing less than females (M = 18.0), and younger people more than older (18–29 (M = 18.3); 30–44 (M = 18.0); 45–59 (M = 18.0); 60+ (M = 16.3), there was no shortage of examples of extensive belief co-endorsements.

Collectively the pattern of belief co-endorsements suggests that while many beliefs are held involving each of the 3 content belief groups, one participant reported holding only one belief (from the SCB group) out of the 46 covered by the CBQ.

**Figure 2:**
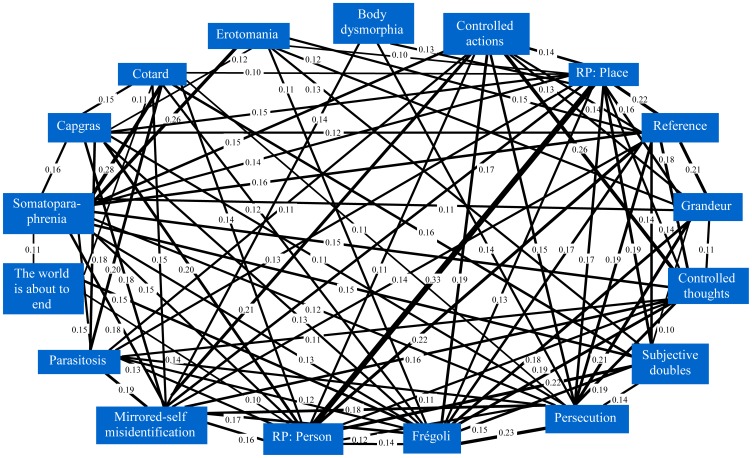
The delusion-like belief pairs with associations of phi (φ) ≥0.1. (RP: Reduplicative paramnesia).

**Figure 3:**
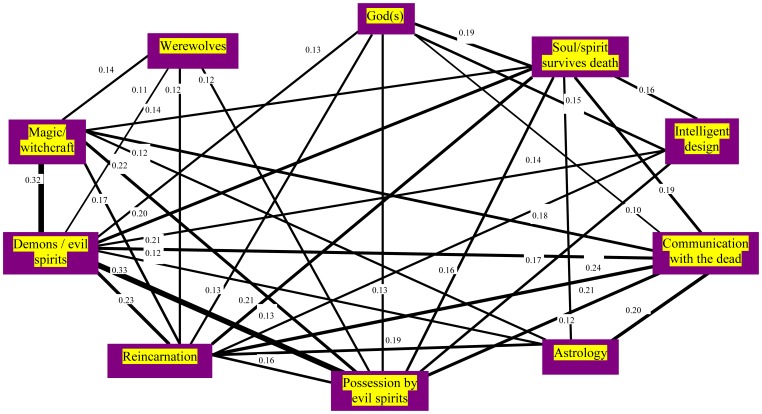
The paranormal and religious belief pairs with associations of phi (φ) ≥0.1.

**Figure 4:**
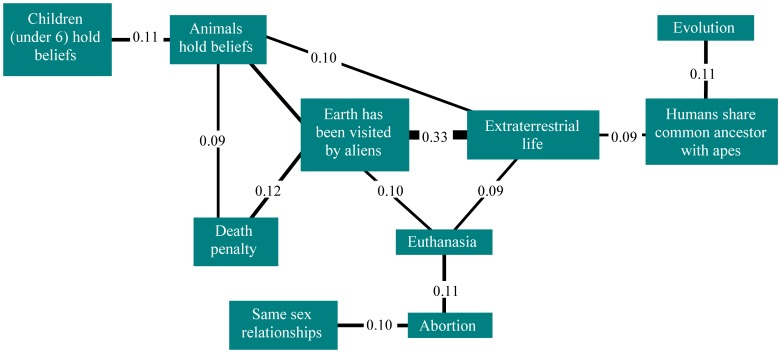
The societal/cultural belief pairs with associations of phi (φ) ≥0.1.

**Figure 5:**
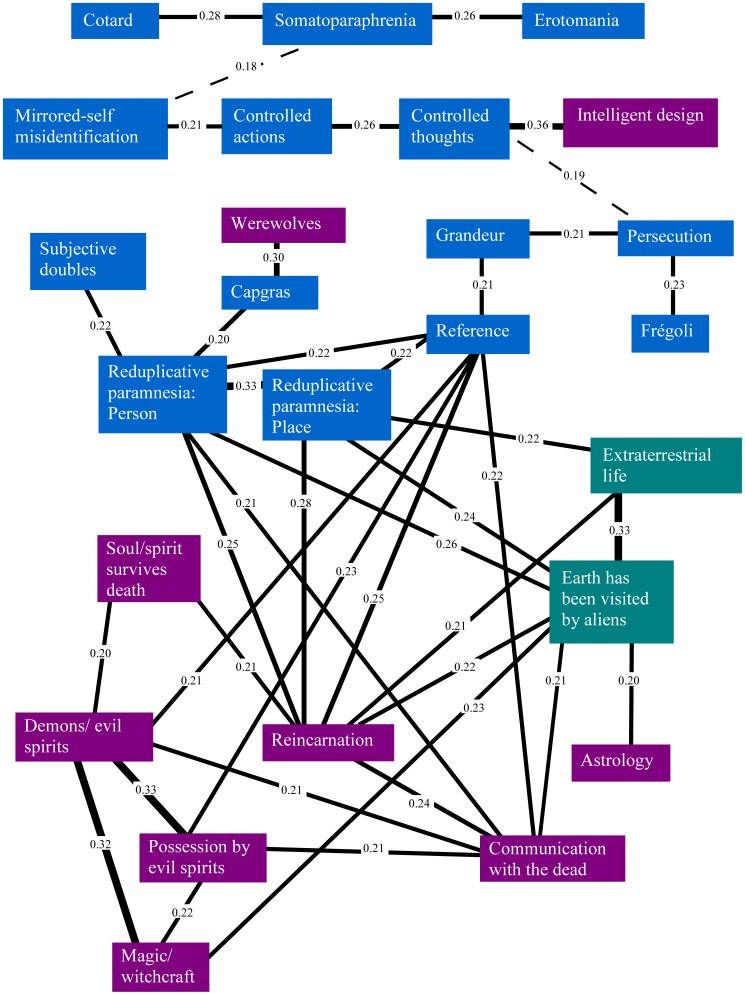
The belief pairs with associations of phi (φ) ≥0.2. Dotted lines indicate the strongest association between members of separated belief groups. These were: Somatoparaphrenia - Mirrored-self misidentification, phi = 0.18; Controlled thoughts - Persecution, phi = 0.19.

Figures 2, 3 and 4 show the significant belief pair associations (all phi>0.1) for the three belief categories. The findings clearly show that delusion-like and paranormal and religious beliefs have much higher co-endorsements than would have been expected by chance alone, and in particular, by comparison to societal/cultural beliefs. Figure 5 shows the strongest of these associations (phi>0.2) for all groups. In general, endorsement of any belief from DLB or P&RB categories (see Figures 2 and 3) provides for a small/moderate increase in the chances of an individual endorsing another belief from that category (as every belief in those categories was significantly associated with at least one other from the same category). However, this was less true for SCB (see Figure 4), where often there were no relationships between endorsements within this heterogeneous belief type, and only one belief pair showed an association of phi (φ) ≥0.2 or more (a pair specifically designed to have similar content: see Figure 4). However, Figure 5 shows that strong associations also crossed between belief categories, especially for paranormal and religious and delusion-like beliefs. In particular, beliefs in reincarnation, aliens visiting Earth, reduplicative paramnesia of both person and place, and ideas of reference were strongly associated with other beliefs including those from other categories.

### 2. Dissonance between Beliefs

Overall, 64.9% of the sample produced consistent belief pairings. 35.1% of the sample produced inconsistent belief pairs: 25.8% of these holding one inconsistent belief pair, 7.7% holding two, 1.6% three or more. Furthermore, 13.1% held strongly inconsistent belief pairs: 11.1% held one inconsistent belief pair, 1.9% held two, and 0.1% three. Table 1 shows the results for individual belief pairings, where the pairing ‘possession by evil spirits’ and ‘demons or evil spirits’ appeared to be the most inconsistent (42% of those endorsing possession not endorsing evil spirits, with 38% still doing so when endorsing possession strongly).

### 3. Demographics

Kruskal-Wallis and Mann-Whitney U tests were used to explore the contribution of demographic variables (Age; Gender; Socioeconomic group; Education; Ethnicity; Religion) to the findings described above for the 5 pairs of beliefs. Significant associations (at p≤0.0001) with the number of inconsistent beliefs were found with older age (χ^2^(3) = 28.59) and lower education (χ^2^(2) = 20.14).

#### Age

Older participants (aged 60+) endorsed significantly more inconsistent belief pairs than those who were younger (aged 18–29 (U(194,269) = 20855.5) or aged 30–44 (U(292,269) = 31723.0)), and there was also a trend towards the 60+ age group having more inconsistent belief pairs than those aged 45–59 (U(245,269) = 27779.0, p = 0.0004). No other age group comparisons were significant.

#### Education

Participants whose highest educational qualification was secondary level showed more inconsistent belief endorsements than those with university qualifications (U(551,274) = 64551.0). However, the comparison between those with a secondary level qualification and those with a higher qualification failed to reach significance; U(551,68) = 15674.0, p = 0.010).

### 4. Discussion

Albeit preliminary, this is the only study that we are aware of that attempts to capture the extent of coherence (or incoherence) between beliefs. As predicted, beliefs within thematic groups were more likely to co-occur more than expected by chance than those between groups. The findings also support previous results [22], indicating that the belief groups with delusion-like, paranormal and religious content comprised items that were more likely to co-occur together, predicted given the strong and reliable association between delusions and paranormal beliefs [23]–[26]. Societal/cultural belief items predictably showed less association with beliefs from the other groups.

Moreover, delusion-like and paranormal and religious beliefs showed more co-occurrences within group than those in the ‘societal/cultural’ group, given the less strong thematic associations within the latter group. Furthermore, this approach allowed for the identification of belief pairs with the strongest associations (with phi>0.3: reduplicative paramnesia of person and of place; possession by evil spirits with demons/evil spirits; possession by evil spirits with magic; extraterrestrial life and aliens having visited Earth), revealing links between those beliefs that might be expected to co-occur.

Contrary to expectations, a larger proportion of individuals reported inconsistent belief pairings although this fell to only 13% when looking at reports of strong beliefs. Interestingly, paranormal belief pairing tended to be more inconsistent than the societal/cultural pair (evolution/sharing a common ancestor with apes). This may be due to these pairs being less formally discussed or fully articulated, with the results that subjects are less likely to be aware of or address discrepancy. Indeed, belief coherence should be stronger for those belief pairs that are more likely to be considered “core” or salient beliefs, and need only hold for those belief pairs where the holder is aware of and which are likely to be those that are more frequently considered. It seems plausible that people may have varying degrees of tolerance for inconsistent beliefs, in particular given the dependence on awareness for one’s beliefs. Indeed, some individuals showed considerable variation in the patterns of belief endorsement. This may be one particularly interesting area of further study when studying individuals with delusional beliefs, to establish whether the degree to which they report dissonant beliefs is different to non-clinical participants. Dysfunctions in evaluating beliefs relative to those beliefs already held by an individual may not in itself be a distinctive feature of delusion formation. Comparisons of the degree to which the beliefs of deluded and non-clinical individuals co-occur or otherwise would however allow evaluation of whether any generic dysfunction in belief evaluation leading to a delusional belief, and/or the selectiveness of this dysfunction, were important factors in the formation or maintenance of pathological beliefs.

Some of the inconsistent beliefs may have arisen due to differences in participants’ interpretation of the question asked, e.g., believing in a certain kind of spirit capable of possession, but thinking of this as distinct from demons, so being wary of endorsing the demons/evil spirits question and not seeing the inconsistency. Indeed, as it is not possible to fully determine how participants interpreted each probe question, this may contribute to some of the apparent discrepancies found in the present study. This limitation, along with a need to replicate the co-occurrence analysis in other sets of belief to ensure that the findings here are not due to the particular selection of belief questions used, provides a strong impetus for further studies in this area. Replication should also include a set of different key belief pairings to further explore reports of seemingly inconsistent beliefs.

It is also important to highlight that co-occurrence of belief endorsement per se could be due to several factors in belief development over and above high level coherence. This might include reasoning biases, which might be expected to impact on the types of beliefs likely to be held. For example, the presence of anomalous experiences (e.g., seeing things) could lead to a number of potentially related beliefs (e.g., in ghosts, spirits, magic, etc). Another limitation is that we cannot identify whether the increased co-occurrence within thematic groups indicates direct coherence between two propositions, or whether this relationship is mediated by one or more other beliefs (and this is likely to vary at the individual level). Ultimately the validity of our conclusions rely on the representativeness of the small sample of beliefs chosen to evaluate each of the three categories.

In conclusion, the degree of belief co-endorsement revealed by this preliminary analysis suggests that endorsing one belief in a thematic group makes it more likely that the same person will endorse another from the group. Although this finding is supportive of ideas arguing for belief coherence, such as those relating to both cognitive consonance [5] and the idea of a web of belief [10], some associations were weaker than might be expected, suggesting that cognitive dissonance (despite subjects not being necessarily aware of same) may also be present. For example, the association between believing in evolution and believing that humans share a common ancestor with apes was relatively weak (φ <0.2) despite the considerable content overlap. The presence of cognitively dissonant beliefs suggest that a strong consistency requirement in defining Quine’s web where beliefs must tie in with all other beliefs, may not be appropriate. While the scale and nature of the items choosen study do not allow us to fully address belief coherence, the results provide a first effort to quantify the "coherence" with which particular beliefs can and are held in the context of other beliefs.
